# Factors in turnover intention of cardiothoracic surgery residents

**DOI:** 10.1038/s41598-023-46588-w

**Published:** 2023-11-20

**Authors:** Ji Hye Yu, Hyun Woong Roh, Mi Ryoung Song, Jang Hoon Lee, Seokjin Haam, Miran Kim

**Affiliations:** 1 Department of Medical Education, Ajou University School of Medicine, Suwon, Korea; 2 Department of Psychiatry, Ajou University School of Medicine, Suwon, Korea; 3 Department of Medical Education, Sungkyunkwan University School of Medicine, Suwon, South Korea; 4 Department of Pediatrics, Ajou University School of Medicine, Suwon, South Korea; 5 Department of Thoracic and Cardiovascular Surgery, Ajou University School of Medicine, 164 Worldcup-Ro, Yeongtong-Gu, Suwon, 16499 South Korea; 6 Department of Obstetrics and Gynecology, Ajou University School of Medicine, 164 Worldcup-Ro, Yeongtong-Gu, Suwon, 16499 South Korea

**Keywords:** Cardiology, Health occupations

## Abstract

Increasing numbers of cardiothoracic surgery residents are resigning, without completing their training. This study analyzes how their turnover intention is related to the training environment, and individual psychological factors. Responses by 57 Korean cardiothoracic surgery residents were analyzed. Their levels of depression, anxiety, grit, and empathy, working conditions, the effect of someone’s presence to discuss their concerns with, burnout, and turnover intention were identified as the research variables. Descriptive statistical analysis, correlation analysis, and structural equation modeling were used for data analysis. Burnout has the most significant relationship with turnover intention. It has a mediating effect on the influence of depression, grit (sustained interest), and working conditions, over turnover intention. Empathy, and the presence of someone to discuss concerns with, also affect turnover intention directly. The study also confirmed that grit and work satisfaction affect turnover intention indirectly, through burnout. The study identified both individual- and systemic-level factors for an effective training environment, to reduce cardiothoracic surgery residents’ tendencies of leaving the residency program, and supporting them for greater satisfaction with their career choice. In order to resolve negative emotions such as burnout and depression, and foster empathy, a human resource development program for the residents’ psychological support must be prepared. The program director should be adequately educated to take charge of the training program, oversee the residents’ education and welfare, and perform the roles of role-model and mentor.

## Introduction

Residency training is one of the health care system’s important processes, for nurturing competent physicians^1^. Thus, leaving the training course midway poses several problems for residents. The resultant loss of transition and patients’ confidence, and an unexpected lack of labor due to resignations, cause problems for the remaining residents throughout the training period^2,3^. It has been found that during the training, many trainees faced psychological health issues such as burnout and depression^4^. The relatively long training programs are the most vulnerable periods for physicians to burnout, considering that these programs entail limited control over circumstances, tremendous responsibilities, excessive and unpredictable work hours, high degree of work-home imbalance, sleep-impairments, and possible mistreatment at the workplace^5–7^. In particular, excessive training causes poor well-being and burnout at work, which ultimately lead to serious considerations of leaving the training^1,8^.

Several studies have shown that psychiatric issues are prevalent among up to 30% of residents, especially among those performing surgery, emergency services, or intensive care rotations^9–12^. Corresponding to this trend, it has been found that cardiothoracic surgery in South Korea faces a major challenge of low numbers of filled residency positions, with a mean filling rate of 45.2% from 2009 to 2018, and the second highest attrition rate of 4.1% from 2017 to 2020^13^. In order to become a cardiothoracic surgeon in South Korea, one has to graduate from medical school, avail a job at a hospital, complete a year-long internship, apply to the department of thoracic and cardiovascular surgery, and then train for four years. Thus, in South Korea, one obtains a specialized certificate five years after graduating from medical school.

Unlike in other countries, in South Korea, one can go for cardiothoracic surgery without a surgical or specialist's license. Although the field’s low filling- and high attrition- rate are global trends, and not limited to South Korea, the need to assess both residency training programs’ situational factors, and cardiothoracic surgery residents’ individual factors, has increased in South Korea^13,14^.

The burnout of physicians and residents is complex in nature^15^. Therefore, a multi-faceted approach is required, not only for measuring burnout, but also assessing other possible mediating factors such as work environment, a reliable training supervisor, symptoms of anxiety and depression, grit personality factor, and subsequent outcomes, such as thoughts of leaving the residency program^16,17^.

Due to the high workload, complexity, high risks of surgery, and increased risks of being involved in a lawsuit, there is a great social reluctance to low participation in the training course for cardiothoracic surgery, which is not limited to Korea^13^. This challenge can result in a high dropout rate of cardiothoracic surgery residents and a persistent low filling rate. Therefore, it is very meaningful to identify variables that affect the turnover intention of cardiothoracic surgery residents.

This study investigated burnout, possible mediators, and subsequent outcomes among cardiothoracic residents in South Korea. Although studies on the phenomenon of residents leaving their programs mid-way are being steadily conducted, it is difficult to locate a study that comprehensively considers how environmental and personal factors affect resignation. The quit intention has great explanatory power for turnover behavior^17^. It helps understand the complex relationship between situational factors, individual factors, tendencies of leaving residency programs, and burnout; the last is widely considered as an influencing factor for turnover intention. The study also considers possible ways of improving residents’ sense of mastery and meaningfulness, during residency training programs^18^.

In this study, factors affecting cardiothoracic surgery residents’ turnover intention were divided into intrapersonal factors and environmental factors, and intrapersonal factors were divided into risk factors and protective factors. In order to find out the effect of each factor on intention to resign, the following research hypothesis was established.

Hypothesis 1 (*H*_1_): The higher the depression and anxiety of cardiothoracic surgery residents, the higher the burnout and intention to resign.

Hypothesis 2 (*H*_2_): The higher the consistency of interest, persistence of effort, and empathy of cardiothoracic surgery residents, the lower the burnout and intention to resign.

Hypothesis 3 (*H*_3_): Cardiothoracic surgery residents’ positive perception of their working conditions and presence of someone to discuss concerns with will reduce burnout and intention to resign.

In this study, three research models were set up and analyzed according to the research hypothesis.

## Methods

### Participants

The study participants included cardiothoracic residents at university hospitals in South Korea. An online survey was conducted with 103 residents, who participated in the Korean Society for Thoracic and Cardiovascular Surgery’s resident training program, in May 2021. The online link to the Google survey was sent on the contact information of all cardiothoracic residents in Korea, with the cooperation of the Korean Society for Thoracic & Cardiovascular Surgery. In order to encourage participation, the questionnaire links were sent three times. Responding to the survey required around 20 min, and a total of 57 people responded; all of them responded to each question diligently, resulting in complete data that could be used for analysis. Informed consent for participation and use of their results was obtained from all subjects. The survey was conducted anonymously. The steps taken for data protection and confidentiality, to ensure secure storage and processing of data, were included in the description provided to them. The study was carried out in accordance with relevant guidelines and approved by the Institutional Review Board (IRB) of Ajou University Hospital (Ethics Consent No. AJIRB-SBR-SUR-20-171, Date of IRB approval: June 23, 2020).

### Measures

To identify the factors behind cardiothoracic residents’ turnover intention, items related to burnout, depression, anxiety, grit, and empathy were considered individual factors, while items related to work environment, and the presence of someone to discuss concerns with, were considered measurement items.

### Residents’ characteristics

The survey considered the participants’ basic demographic items such as gender, age, year of training, and location of training hospital.

### Burnout

The validated 22-item Maslach Burnout Inventory-Human Services Survey (MBI-HSS) was used to measure burnout^19^. It comprises of three sub-scales for evaluating three domains of burnout: emotional exhaustion (EE), depersonalization (DP), and reduced personal achievement (PA). Each item was scored on a 7-point Likert scale, with response options ranging from “never” to “always.” Higher scores indicate frequent occurrences of burnout. Cronbach’s α for the scale was 0.875.

### Depression

The 21-item Korean-Beck Depression Inventory-II (K-BDI-II) was used to measure depression^20^. Each item was scored on a 4-point Likert scale, from “never” to “very often,” with higher scores representing higher levels of depression. Cronbach’s α for the scale was 0.882.

### Anxiety

The 21-item Korean-Beck Anxiety Inventory (K-BAI) was used to measure anxiety^21,22^. Each item was scored on a 4-point Likert scale, from “not all” to “severely,” with higher scores representing higher levels of anxiety. Cronbach’s α for the scale was 0.911.

### Grit

The eight-item short grit scale (Grit-S) was used to measure perseverance and passion for pursuing long-term goals^23^. The scale has two components: persistence of effort, and consistency of interest. Each item was scored on a 5-point Likert scale, from “never” to “strongly agree,” with higher scores representing greater passion and patience for achieving long-term goals. Cronbach’s α for the scale was 0.788.

### Empathy

The 20-item Jefferson Scale of Physician Empathy-Health Professional (JSPE-HP) was used for measuring empathy^24^. The scale has three components: perspective taking, compassionate care, and standing in a patient’s shoes. Each item was scored on a 7-point Likert scale, from “never” to “strongly agree,” with higher scores representing higher levels of empathy. Cronbach’s α for the scale was 0.851.

### Working conditions

A scale developed by Wada et al.^25^ was used for assessing working conditions in the training hospitals. The scale consists of 29 items related to personal time, relationships with patients, other physicians and staff, patient-care issues, administrative work, income, resources, job satisfaction, and workload. This study measured each item on a 5-point Likert scale, ranging from “never” to “strongly agree,” with higher scores representing better working conditions. Cronbach’s α for the scale was 0.868.

### Presence of someone to discuss concerns with

The presence of someone to discuss concerns with was assessed by the question, “When you have difficulties or concerns, is there anyone in the hospital you can discuss them with?” Participants were asked to respond with 1 (yes) or 2 (no).

### Turnover intention

The degree of turnover intention was measured by the question, “Have you ever considered resigning during the training period?” Responses were measured on a 5-point Likert scale, from “never” to “strongly agree.”

### Statistical analysis

All analyses were conducted using JAMOVI 1.8.2.0. Descriptive statistics were calculated for the demographic, as well as other research variables. Then, a correlation analysis was conducted for exploring the relationship between variables related to turnover intention. In addition, a structural equation model was confirmed for the structural relationships among individual factors, working conditions, and turnover intention.

### Ethical approval

This study was approved by the Institutional Review Board (IRB) of Ajou University Hospital (Ethics Consent No. AJIRB-SBR-SUR-20–171, Date of IRB approval: June 23, 2020).

### Informed consent

Consent was obtained from all the study participants.

## Results

### Demographic characteristics

**Figure 1:**
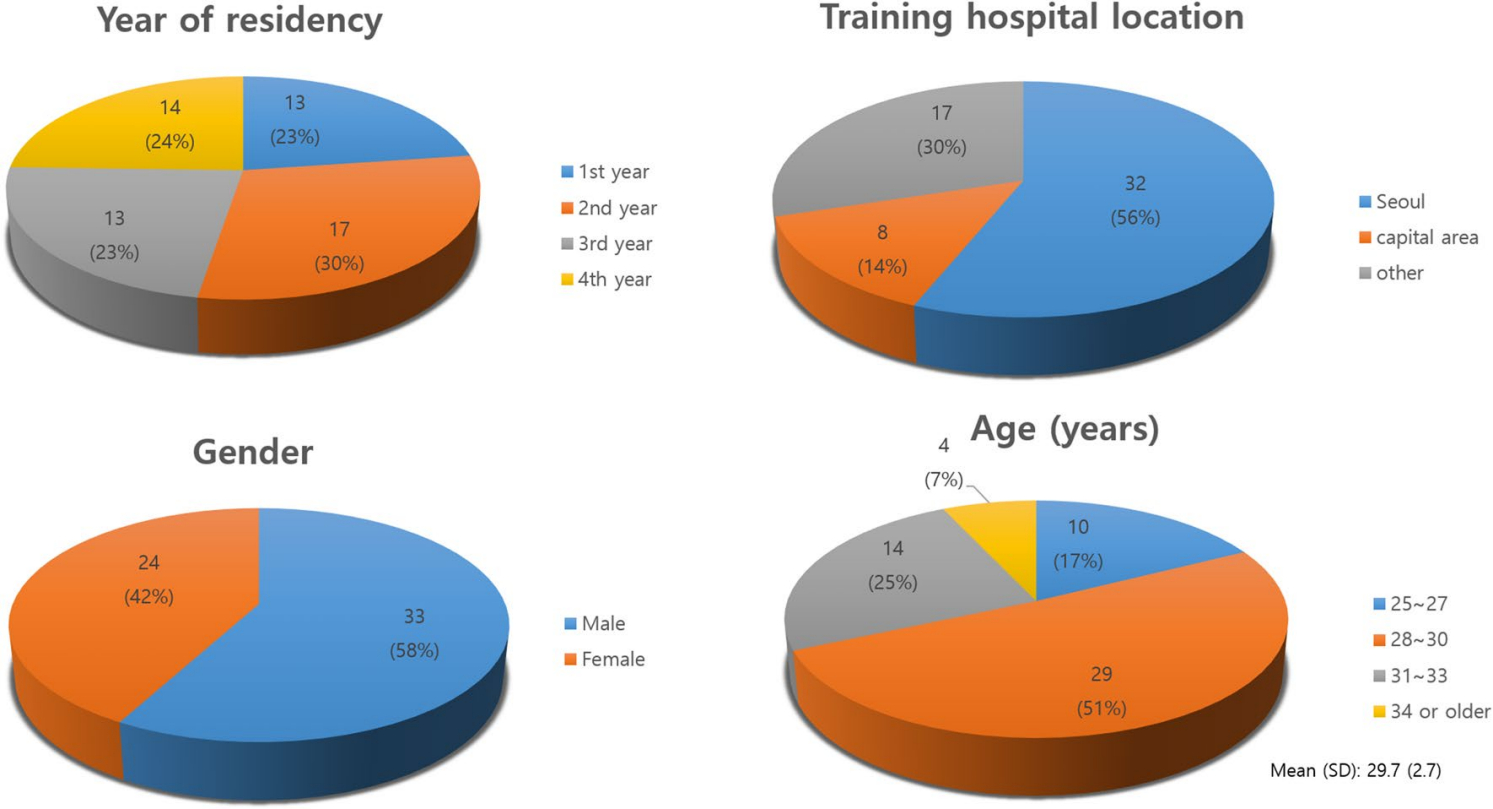
Socio-demographics of cardiothoracic surgery residents’ sample (N = 57).

The survey was completed by 57 participating residents. Their demographic characteristics are summarized in Fig. 1. Participants demonstrated a relatively even distribution, in terms of gender and year of residency. More than half of the participants had undergone training in hospitals located in Seoul. The average age of the respondents was 29.7 years.

### Descriptive and correlation statistics of study variables

The descriptive statistics of, and correlations among, the variables are presented in Table 1. Their skewness and kurtosis values were checked to verify a normal distribution; the absolute value of skewness did not exceed 3, and that of kurtosis did not exceed 10^26^.

**Table 1:** Descriptive and correlation statistics of study variables.

	1	2	3	4	4_1	4_2	5	6	7
1. Turnover intention	—								
2. Burnout	0.63***	—							
3. Empathy	−0.26	−0.04	—						
4 Grit	−0.18	−0.09	0.34**	—					
4_1. CI	−0.27*	−0.25	−0.15	0.31*	—				
4_2. PE	−0.27*	−0.09	0.36**	0.91***	0.27*	—			
5. Depression	0.51***	0.52***	−0.17	−0.33*	−0.43***	−0.30*	—		
6. Anxiety	0.40**	0.5***	−0.02	−0.24	−0.37**	−0.20	0.67***	—	
7. Working condition	−0.43***	−0.42**	0.3*	0.27*	0.29*	0.38**	−0.54***	−0.32*	—
8.Presence of someone to discuss concerns with	−0.39**	−0.21	0.21	0.16	0.45***	0.18	−0.48***	−0.27*	0.08
M(SD)	2.54(1.13)	4.46 (0.56)	5.04 (0.64)	3.05 (1.03)	3.50 (0.70)	2.97 (0.83)	9.7 (6.95)	8.3 (7.94)	3.34 (0.44)
Skewness (< 3)	0.15	0.39	0.25	0.41	−0.23	0.05	1.16	2.05	−0.62
Kurtosis (< 10)	−0.85	0.06	−0.46	−0.70	−1.03	−0.40	1.75	6.45	2.58

CI = Consistency of interest; PE = Persistence of effort.

### Structural relationships among individual variables, working conditions, burnout, and turnover intention

This study considered burnout as the major variable in intentions of resigning, and established a structural equation model for analyzing the structural relationship between individual and situational variables affecting turnover intention. A model was established with the individual factors, by dividing the variable that positively affects turnover intention and burnout, and the variables that negatively affect grit. Among the individual variables, those that affect turnover intention and burnout positively and negatively were classified, and accordingly, structural models were designed.

### Relationships among depression, anxiety, burnout, and turnover intention

**Figure 2:**
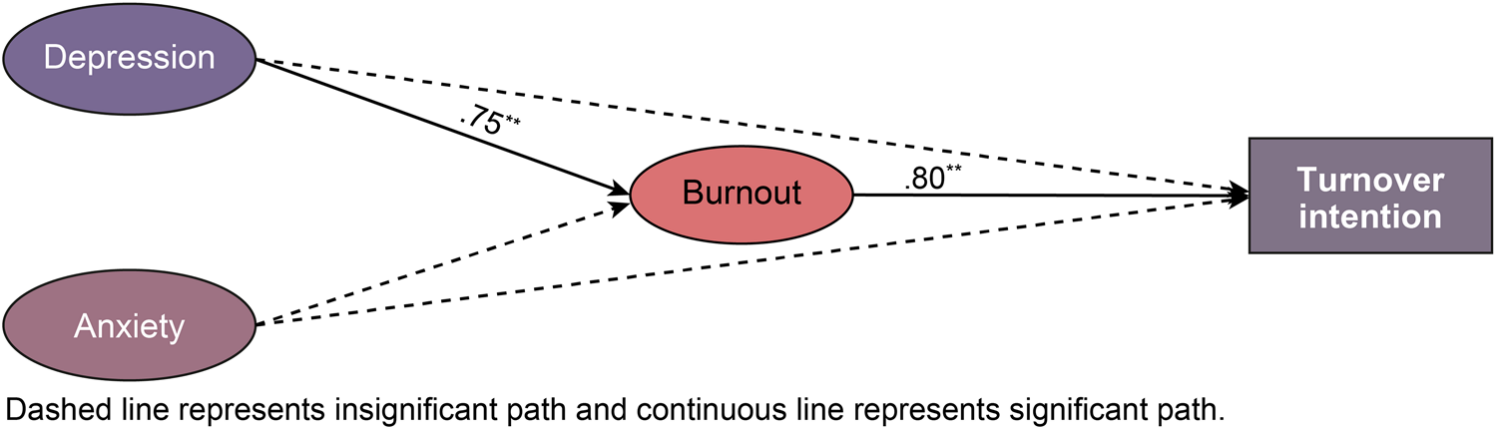
Structural model of depression, anxiety, burnout, and turnover intention.

Figure 2 presents the structural model of relationships among depression, anxiety, burnout, and turnover intention. The model fit was χ^2^ = 23.87 (df = 23, *p* < 0.05), χ^2^/df = 1.15, CFI = 0.98, TLI = 0.97, RMSEA = 0.05 (90% CI 0.00–0.11), which is considered adequate.

The path coefficients between the structural model’s variables are listed in Table 2. Among the individual factors, depression has a significant positive effect on burnout (β = 0.75, *p* < 0.01), and in turn, burnout has a significant positive effect on turnover intention (β = 0.80, *p* < 0.01). The bootstrap confidence interval was used to verify the mediating effect’s statistical significance, based on the meaningful path. It was found that the higher a resident’s level of depression, the greater was the exhaustion. This, in turn, heightened their intention of resigning, thereby confirming the significance of an indirect effect (β = 0.60, 95% CI [0.11, 2.36]).

**Table 2:** Path coefficients of the structural model of relationships among depression, anxiety, burnout, and turnover intention.

**Direct effects**	B	β	S.E	C.R
Depression → burnout	2.63	.75	.94	2.81**
Anxiety → burnout	.75	.09	2.03	.37
Burnout → turnover intention	.98	.80	.33	2.99**
Depression → turnover intention	.65	.15	1.27	.51
Anxiety → turnover intention	2.10	.21	2.27	.93

Indirect effects	B	β	Bootstrapped 95% CI	
depression → burnout → turnover intention	2.58	.60	.11–2.36	
anxiety → burnout → turnover intention	.74	.07	−.78–0.72	

Model fit: χ^2^ = 23.87(df = 23, *p* < .05), χ^2^/df = 1.15 CFI = .98, TLI = .97, RMSEA = .05 (90% CI .00–.11).

S.E. = Standard Errors; C.R. = Critical Ratio.

**p < .01, ***p < .001;

Number of bootstrap samples = 2000;

LL = lower limits; UL = upper limits; CI = confidence interval.

### Relationships among grit, empathy, burnout, and turnover intention

**Figure 3:**
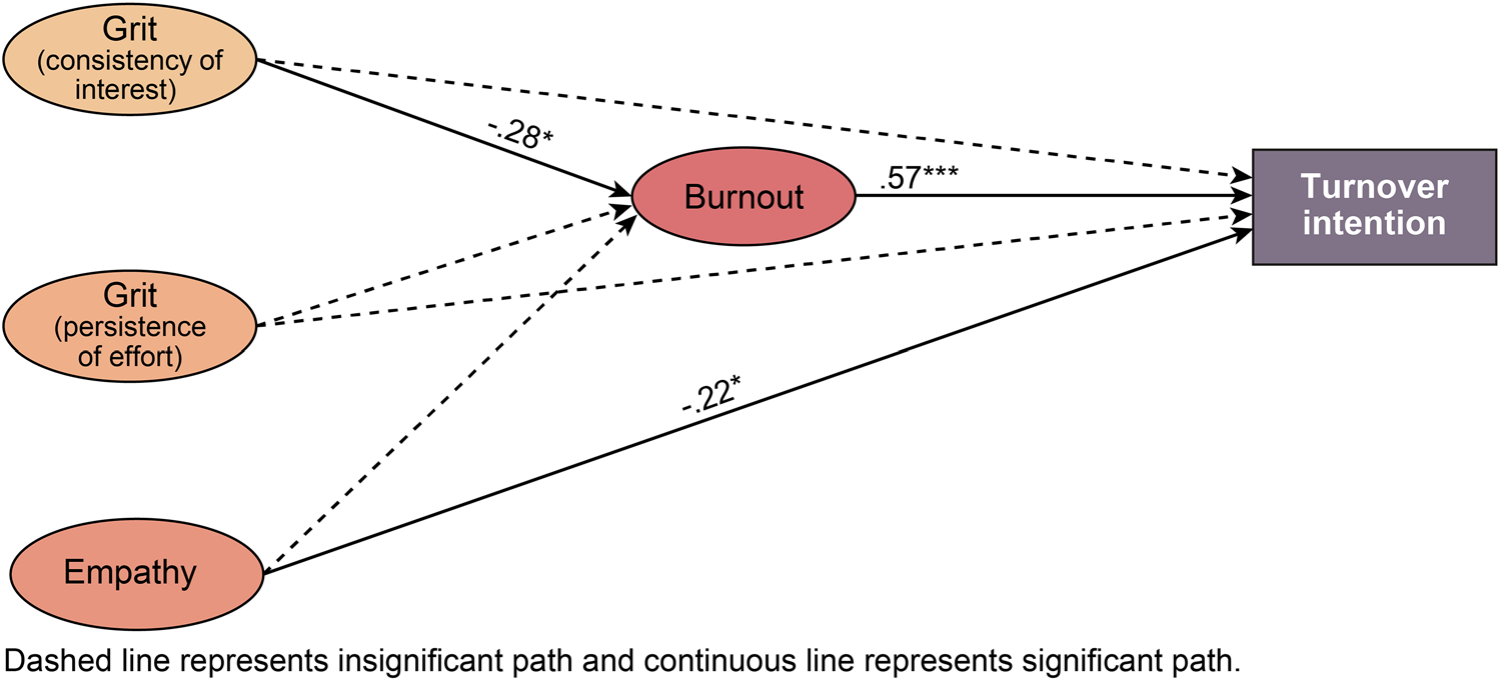
Structural model of grit, empathy, burnout, and turnover intention.

Figure 3 presents a structural model of the relationships among grit, empathy, burnout, and turnover intention. The model fit was χ^2^ = 13.72(df = 10, *p* < 0.001), χ^2^/df = 1.37, CFI = 0.95, TLI = 0.93, RMSEA = 0.08 (90% CI 0.00–0.18), which is considered adequate.

The path coefficients between the structural model’s variables are listed in Table 3. Consistency of interest, which is a sub-factor of grit, has a significant negative effect on burnout (β = −0.28, *p* < 0.05), and in turn, burnout has a significant positive effect on turnover intention (β = 0.57, *p* < 0.001). In addition, empathy has a significant negative effect on turnover intention (β = −0.22, *p* < 0.05).

**Table 3:** Path coefficients of the structural model of relationships among grit, empathy, burnout, and turnover intention.

**Direct effects**	B	β	S.E	C.R
Grit (consistency of interest) → burnout	−.25	−.28	.11	−1.98*
Grit (persistence of effort) → burnout	.01	.01	.10	.10
Empathy → burnout	−.07	−.08	.12	−.57
Burnout → turnover intention	1.19	.57	.20	5.84***
Grit (consistency of interest) → turnover intention	−.21	−.13	.17	−1.22
Grit (persistence of effort) → turnover intention	−.14	−.10	.15	−.89
Empathy → turnover intention	−.40	−.22	.19	−2.12*

Indirect effects	B	β	Bootstrapped 95% CI	
grit (consistency of interest) → burnout → turnover intention	−.25	−.16	−.35 to −.02	
grit (persistence of effort) → burnout → turnover intention	.01	.01	−.19–.20	
empathy → burnout → turnover intention	−.08	−.05	−.21–.11	

Model fit: χ^2^ = 13.72 (df = 10, *p* < .001), χ^2^/df = 1.37, CFI = .95, TLI = .93, RMSEA = .08 (90% CI .00 − .18).

S.E. = Standard Errors; C.R. = Critical Ratio.

By confirming the mediating effect’s statistical significance, it was found that as grit (consistency of interest) decreased, burnout increased, which heightened intentions of resigning, and confirmed the significance of burnout’s indirect effect (β = −0.16, 95% CI [−0.35, −0.02]).

### Relationships among working conditions, presence of someone to discuss concerns with, burnout, and turnover intention

**Figure 4:**
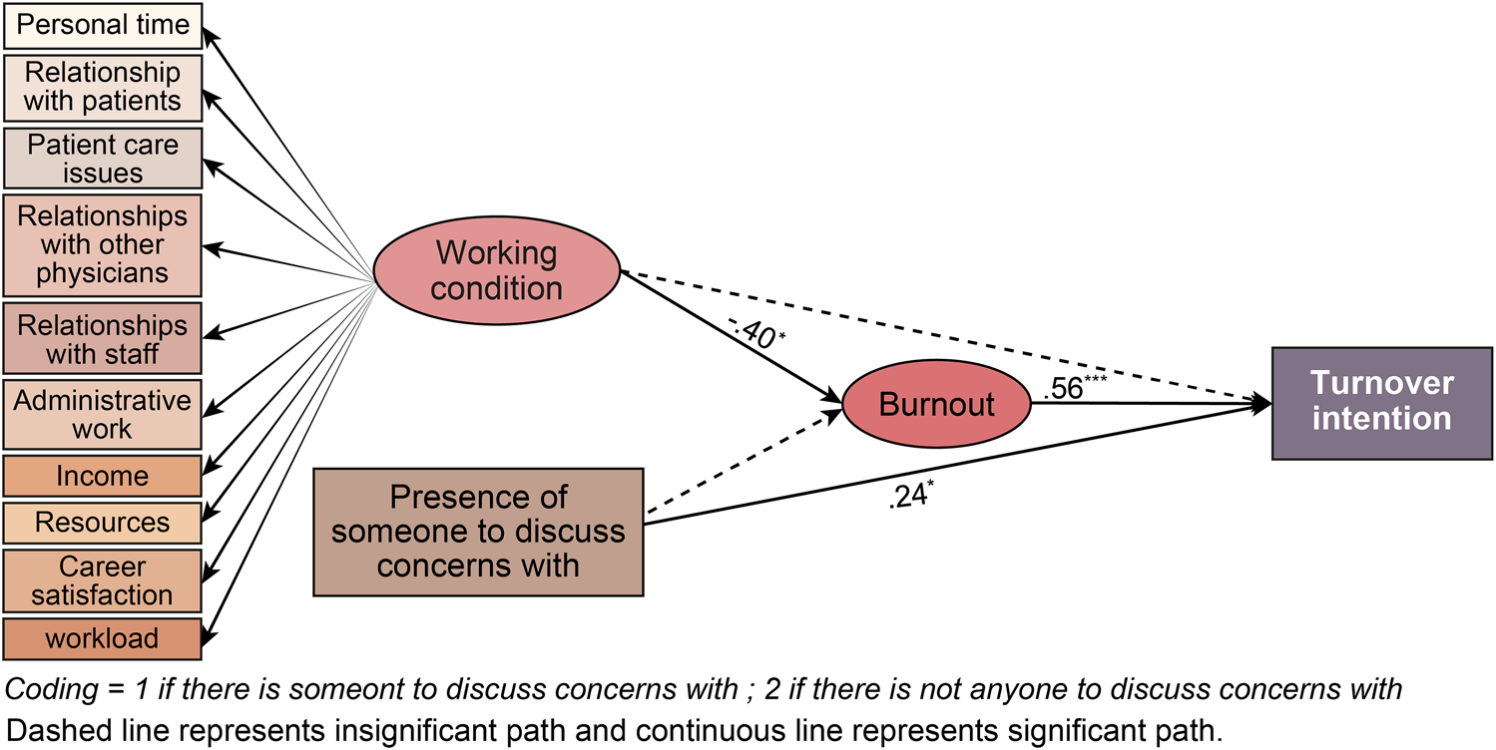
Structural model of working conditions, presence of someone to discuss concerns with, burnout, and turnover intention.

Figure 4 presents a structural model of the relationships among working conditions, presence of someone to discuss concerns with, burnout, and turnover intention. The model fit was χ^2^ = 77.133 (df = 58, *p* < 0.05), χ^2^/df = 1.33, CFI = 0.90, TLI = 0.86, RMSEA = 0.08 (90% CI 0.01–0.12), which is considered adequate.

Table 4 presents the structural model’s path coefficients. Working conditions have a negative effect on burnout (β = −0.40, *p* < 0.05), and in turn, burnout has a positive effect on turnover intention (β = 0.56, *p* < 0.001). Further, the lack of someone in the hospital to discuss concerns with heightened intentions of resigning (β = 0.24, *p* < 0.05); however, this presence or absence did not affect burnout significantly. In addition, it was confirmed that the indirect effect of burnout as a mediating variable was significant in the relationship between working conditions and turnover intention (β = −0.22, 95% CI [−0.44, −0.03]).

**Table 4:** Path coefficients of the structural model of relationships among working condition, presence of someone to discuss concerns with, burnout, and turnover intention.

Direct effects	B	β	S.E	C.R
Working condition → burnout	−.32	−.40	.14	−2.24*
Presence of someone to discuss Concerns with → burnout	−.01	−.01	−.06	.96
Burnout → turnover intention	1.14	.56	.22	5.16***
Working condition → turnover intention	−.10	−.06	.24	−.40
Presence of someone to discuss concerns with → turnover Intention	.75	.24	.37	2.02*

Indirect effects	B	β	Bootstrapped 95% CI	
Working condition → burnout → turnover intention	−.85	−.22	−.44 − −.03	
Presence of someone to discuss concerns with → burnout → turnover intention	−.57	−.01	−.18 − .13	

Model fit: χ^2^ = 77.133(df = 58, *p* < .05), χ^2^/df = 1.33, CFI = .90, TLI = .86, RMSEA = .08 (90% CI .01 − .12).

S.E. = Standard Errors; C.R. = Critical Ratio.

*p < .05, ***p < .001;

Number of bootstrap samples = 2000;

LL = lower limits; UL = upper limits; CI = confidence interval.

The test results from *H*_1_ to *H*_3_ set in this study are summarized as follows.

H1 (The higher the depression and anxiety of cardiothoracic surgery residents, the higher the burnout and intention to resign.): The higher the depression, the higher the burnout, and in turn, the higher the burnout, the higher the turnover intention. Anxiety was found to have no statistically significant effect on burnout and turnover intention.

H2 (The higher the consistency of interest, persistence of effort, and empathy of cardiothoracic surgery residents, the lower the burnout and intention to resign.): The higher the consistency of interest, the lower the burnout, and in turn, the lower the burnout, the lower the turnover intention. Persistence of effort was found to have no significant effect on burnout and turnover intention. Empathy was found to have no significant effect on burnout, but it was found to have a negative effect on turnover intention, indicating that the higher the level of empathy in the patient-physician relationship, the lower the intention to resign.

H3 (Cardiothoracic surgery residents’ positive perception of their working conditions and presence of someone to discuss concerns with will reduce burnout and intention to resign.): A positive perception of working conditions lowered burnout, which sequentially lowered the intention to resign. And the presence of someone to discuss concerns with was found to have no significant effect on burnout, but the absence of someone to discuss concerns was found to increase the intention to resign.

## Discussion

This study analyzed the effects of individual and environmental factors on South Korean cardiothoracic surgery residents’ turnover intention. The results reveal that burnout has the highest positive correlation with turnover intention. Previous studies have also found burnout to be a major variable in increased turnover intention^2,27–29^. Surgeons and trainees were particularly reported as being more frequently burnt out than others. Thus, cardiothoracic surgery residents can be considered quite vulnerable to burnout^4,30^. Reducing their burnout would lower turnover intention.

It is necessary to reduce the risk factors for burnout, and strengthen the protective factors against it. The first step is identifying the factors that cause burnout. Among the individual factors, depression was found to have a significant effect on turnover intention, by mediating burnout. The higher the residents’ level of depression, the greater was the degree of burnout, in turn leading to heightened intentions of resigning. This can be understood within the same context as that of a study by Chaukos et al.^31^, which shows that burnt out residents showed more depressive symptoms, than those who were not burnt out. The present findings confirm that reducing depression among residents is necessary to lower burnout and turnover intention.

In this research model, anxiety was found to have no significant effect on burnout, but anxiety, along with depression, appeared to be an intrapersonal variable with the highest correlation with burnout. However, anxiety showed a lower correlation with burnout than depression. In a previous study that analyzed the relationship between burnout, depression, and anxiety, similar to the results of this study, both depression and anxiety were found to have a significant association with burnout, but anxiety had a lower correlation with burnout than depression^32^. Since anxiety has a relatively lower correlation with burnout than depression, it can be inferred that when anxiety and depression are included together in the research model, anxiety does not have a significant effect on burnout because its influence on burnout is relatively small. However, in this study, anxiety had a significantly high correlation with depression and burnout, so it is necessary to consider the level of anxiety of cardiothoracic surgery residents and introduce a practical program to reduce anxiety.

This study also found that grit (consistency of interest) affects turnover intention, by mediating burnout. A resident doctor who can maintain interest in the task to be accomplished experiences less burnout, which lowers the intention to resign. This can be understood in the same context as that of a previous study^17^ on emergency medicine residents, which finds that burnt out residents scored significantly lower on grit, than those who were not burnt out. Furthermore, empathy was found to have a significant positive effect on turnover intention, without mediating burnout. This suggests that the greater the empathy in patient-doctor relationships, the lower is the intention to resign. Studies on the relationship between empathy and burnout have contradictory results; however, most studies report that empathy and burnout have a negative relationship^33^.

Working conditions, including personal time, relationships with others (patients, physicians, staffs), patient-care issues, administrative work, income, resources, job satisfaction, and workload, were found to have a significant effect on turnover intention, by mediating burnout. Job satisfaction and positive perception of working conditions leads to less burnout, which in turn, lowers turnover intention. Studies on healthcare professionals^27,29,34^ report that high levels of burnout are related to increased work pressure, and less satisfaction with one’s work and workspace; this is consistent with the present study’s results. In addition, this study found that the presence or absence of someone inside the hospital, to discuss concerns with, has a direct significant effect on turnover intention, without mediating burnout. The intention to resign is relatively low when there is someone in the hospital to discuss concerns with, as compared to when there is no one. A well-organized mentoring system is essential and recommended for residents to adapt well during training course, be satisfied with training programs, and have successful career development^35^ Mentorship is recognized as an important factor in increasing interest in specialty and preventing burnout during the training process, and is also associated with retention^36,37^. Previous studies have shown that formal education on mentorship and programs for developing mentorship skills are insufficient in cardiothoracic surgical training^36^. Formal education on mentorship should be provided at the individual level and at the institutional level to effectively support cardiothoracic surgery residents during the training process. Social support is a decisive factor in employees’ engagement in an organization^38^, and provides a context for understanding the present study’s results. This study is significant for its empirical investigation of the relationship between variables affecting turnover intention of cardiothoracic surgery residents. The results confirm the importance of reducing burnout, improving empathy, and having someone in the hospital to discuss concerns with, for reducing residents’ turnover intention, and nurturing medical professionals specializing in cardiothoracic surgery. To particularly reduce burnout, which is highly related to turnover intention, it is necessary to diagnose psychological issues such as depression, and provide necessary support. Moreover, the working environment in cardiothoracic surgery departments also needs improvement. In addition, residents’ satisfaction with their field, and interest in work, can also help reduce burnout.

This study has certain limitations, which should be noted. First, the sample size was small. It included around 56% of all cardiothoracic surgeons in Korea. Therefore, it is difficult to draw generalizations from it. However, as important as the size of the survey sample, is the presence of biases in sampling. This study’s sample did not concentrate on a specific gender, year of training or age, rather, it was a relatively even sample. This may somewhat compensate for the small sample size. Second, the survey was conducted during the COVID-19 pandemic. Therefore, psychological and environmental variables related to COVID-19 might have influenced the survey responses, but the study’s analysis did not consider this. Third, the survey was conducted via self-reporting questionnaires. In the future, qualitative research methods can be employed for in-depth understanding of the factors influencing intentions of resigning. Fourth, this study was cross-sectional, and therefore, could not confirm changes in related variables such as turnover intention and job burnout, throughout the residents’ training period. Future research could comprise of longitudinal studies for determining causal relationships between turnover intention and related variables.

## Conclusion

Cardiothoracic surgery residents’ individual and environmental variables affect their turnover intention, either directly or indirectly. Among the individual variables, burnout and empathy heightened and lowered their intentions of resigning from the program, respectively. In addition, it was confirmed that residents’ depression and grit (consistency of interest) have an indirect effect on turnover intention, through burnout. Among the situational variables, the presence of someone in the hospital to discuss concerns with has a direct positive effect on turnover intention, while satisfaction with the working environment mediated burnout and affected turnover intention.

In order to resolve negative emotions such as burnout and depression, and foster empathy, a human resource development program for residents’ psychological support must be prepared. The program director should be adequately educated to take charge of the training program, oversee the residents’ education and welfare, and perform the roles of role-model and mentor.

## Data Availability

The datasets used and/or analyzed during the current study available from the corresponding author on reasonable request.

## Abbreviations

MBI-HSS: Maslach Burnout Inventory-Human Services Survey
EE: Emotional exhaustion
DP: Depersonalization
PA: Personal accomplishment
K-BDI-II: Korean-Beck Depression Inventory-II
K-BAI: Korean-Beck Anxiety Inventory
Grit-S: Short Grit Scale
CI: Consistency of interest
PE: Persistence of effort
JSPE-HP: Jefferson Scale of Physician Empathy-Health Professional
